# A Church-Based Weight Loss Intervention in African American Adults using Text Messages (LEAN Study): Cluster Randomized Controlled Trial

**DOI:** 10.2196/jmir.9816

**Published:** 2018-08-24

**Authors:** Robert L Newton Jr, Leah A Carter, William Johnson, Dachuan Zhang, Sandra Larrivee, Betty M Kennedy, Melissa Harris, Daniel S Hsia

**Affiliations:** ^1^ Pennington Biomedical Research Center Baton Rouge, LA United States

**Keywords:** African Americans, behavioral strategies, community health, mHealth, mobile phone, obesity, text messages, weight loss

## Abstract

**Background:**

African American adults experience a high prevalence of obesity and its associated comorbidities, including diabetes. Church-based interventions have been shown to be effective in decreasing weight in this population. mHealth interventions can address two needs for obesity treatment in this community, including enhancing weight loss and providing wide dissemination.

**Objective:**

This study aimed to assess the feasibility and efficacy of a church-based weight loss intervention that incorporates mHealth technology.

**Methods:**

In this study, 8 churches (n=97) were randomly assigned to the intervention or delayed intervention condition (control group). We recruited participants through their respective church. Volunteer church members were trained by study staff to deliver the 10-session, 6-month intervention. Participants in the intervention group attended group sessions and received automated short message service (SMS) text messages designed to reinforce behavioral strategies. Conversely, participants in the delayed intervention condition received SMS text messages related to health conditions relevant for African American adults. We obtained measures of body composition, blood pressure, blood glucose, and cholesterol.

**Results:**

We successfully recruited 97 African American adults, with a mean age of 56.0 (SE 10.3) years and a mean body mass index of 38.6 (SE 6.4) kg/m2 (89/97, 91.8% females), who attended the churches that were randomized to the intervention (n=68) or control (n=29) condition. Of these, 74.2% (72/97) of the participants (47/68, 69.1% intervention; 25/29, 86.2% delayed intervention) completed the 6-month assessment. The average intervention group attendance was 55%. There was a significant difference in weight loss (*P*=.04) between participants in the intervention (–1.5 (SE 0.5) kg) and control (0.11 (SE 0.6) kg) groups. Among participants in the intervention group, the correlation between the number of SMS text messages sent and the percent body fat loss was r=.3 with *P*=.04. The participants reported high satisfaction with the automated SMS text messages.

**Conclusions:**

Automated SMS text messages were well-received by participants, suggesting that more enhanced mHealth technologies are a viable option for interventions targeting African American adults.

**Trial Registration:**

ClinicalTrials.gov NCT02863887; https://clinicaltrials.gov/ct2/show/NCT02863887 (Archived by WebCite at http://www.webcitation.org/71JiYzizO)

## Introduction

Obesity represents a health inequity for African American adults [1,2]. African American adults with obesity are at greater risk for developing chronic diseases such as diabetes. A major risk factor for developing obesity and its comorbidities is an unhealthy lifestyle comprising a lack of regular physical activity and poor nutrition [3,4]. Intensive behavioral programs have resulted in improved dietary intake, increased physical activity, and weight loss [5-7]. However, weight loss results for African American adults have been less than those for other ethnic groups [8]. Therefore, innovative strategies addressing the obesity epidemic in African American adults are warranted.

One novel strategy could be the utilization of mHealth technology (ie, mobile phones, wearables, etc). Mobile phones are an emerging intervention tool because they are compact, portable, normally “on,” readily available to individuals, affordable, user-friendly, offer advanced functionality, and allow Web-based access 24 hours a day for 7 days per week [9-11]. mHealth technologies can affect the amount of treatment delivered by increasing communication between study staff and participants, providing behavioral strategies, and promoting behavioral change [12]. Several reviews [13-16] have shown that mHealth interventions can be effective in promoting weight loss; however, few of these studies have included substantial numbers of African American adults despite this ethnic group’s well-documented high prevalence of obesity.

mHealth interventions utilizing mobile phones may be particularly well suited for African American adults. Recent estimates have shown that 94% of African American adults own a mobile phone [17], 72% own a smartphone [17], and nearly 70% utilize mobile phones to access health information [18]. Short message service (SMS) text messaging is one form of mHealth, and African American adults report that SMS text messaging is an appropriate form of intervention delivery [19,20]. This realization has led to the development of SMS text message-based pilot and feasibility studies targeting various behaviors such as the medication adherence, HIV prevention, healthy eating, and physical activity [21-25]. Although these studies demonstrate the acceptability, feasibility, and potential effectiveness of SMS text message interventions designed for African American adults, few have utilized randomized controlled designs [22], and fewer were specifically designed to promote weight loss [26]. Therefore, the purpose of the Lifestyle Changes Through Exercise and Nutrition (LEAN) study was to test the feasibility (SMS text message tolerance and satisfaction) and effectiveness (compared with a control group) of utilizing SMS text messages to promote weight loss in African American adults enrolled in a church-based weight loss program.

## Methods

### Design

The study utilized a cluster-randomized trial design (NCT02863887). Originally, 9 churches agreed to participate and were randomized into the study. While 5 churches were randomized to the intervention group, 4 churches were randomized to the control group. Notably, the churches were matched by their membership size between the conditions so that the potential participant sample sizes would be similar.

### Participants

Individuals were considered eligible for this study if they (1) were aged 18-75 years, (2) self-identified as African American, (3) were a member of a participating church, (4) had a body mass index (BMI) of >30 kg/m^2^, (5) owned a mobile phone with SMS text messaging capabilities, (6) were at risk for diabetes (ie, had a history of gestational diabetes or at least one diagnosed nuclear family member) or had been diagnosed with type 2 diabetes, (7) were willing to change their diet and physical activity to promote weight loss, (8) were able to participate in face-to-face counseling sessions as scheduled, and (9) had low to moderate cardiovascular disease risk. Participants were paid up to US $60 for completing the study.

### Procedures

Churches were recruited utilizing a community outreach coordinator who attended pastoral fellowship meetings and held an initial meeting with the pastor or health ministry leader. Then, the participating churches were randomized to either the intervention or control group. Churches were randomized to the intervention or control group using a 1:1 ratio; two churches with the largest and smallest memberships (n=8000 and 75) were clustered together. The study statistician used a computerized pseudorandom number generator to determine the randomization order in advance. The study coordinator revealed the randomization to the churches prior to the start of the study. Following randomization, participants were recruited utilizing standard strategies, including presentations during church service, small groups, and health fairs; flyers placed on informational boards, tables, etc; advertisements placed in the church bulletins; and utilizing the community health coaches from each church who volunteered to deliver the intervention (refer to the Community Health Coaches section below). Next, interested individuals were phone-screened to determine their initial eligibility. Those eligible were invited to the baseline visit. During the baseline visit, potential participants were provided with information related to the purpose, setting, and duration of the program; the nature of the behavioral change program; information on the number, duration, timing, and content of the automated SMS text messages; participant and community health coach expectations; and the assessments. Written informed consent was obtained from each potential participant before they completed their baseline assessments. Baseline assessments and community health coach training were completed approximately 1 week prior to the start of the intervention. This study protocol was reviewed and approved by the Pennington Biomedical Research Center’s Institutional Review Board.

### Study Groups

#### Intervention

##### Community Health Coaches

Each church was required to select 2 volunteers from their congregation to lead the group sessions. Although these individuals are often referred to as “lay health providers,” the volunteers in the LEAN study collectively decided to be referred to as “community health coaches.” All community health coaches were women who were employed as nurses, pharmacists, or health education or physical education teachers. Community health coaches received an 8-hour training conducted by the study staff on how to deliver a behaviorally based, empirically proven, lifestyle change and weight loss intervention (ie, Diabetes Prevention Program). Several topics were covered during the training, including leading effective groups, behavior modification, physical activity demonstrations, and a detailed review of each session’s content.

##### Group Sessions

At each designated church, 10 group sessions (Textbox 1) were conducted over a 6-month period. The session frequency was weekly (n=4) for the first month, bimonthly for 2 months (n=4), and monthly for 2 months (n=2). Attendance and participant weight were recorded at each session by the community health coach.

##### Short Message Service Text Messages

Participants received 5 automated SMS text messages each week pertaining to lesson content (n=2), motivation (n=1), and behavioral prompts (n=2). Lesson content SMS text messages contained information derived from the lesson materials (ie, behavioral strategies, information, reminders, etc). Motivational SMS text messages contained verbiage that encouraged and reinforced participant behavioral change. Behavioral prompt SMS text messages were designed to remind participants to engage in physical activity, check weight daily, and consume healthy meals. A library of SMS text messages was created for each component, and each participant received a random selection of these messages. These SMS text messages were adapted from a previous physical activity intervention conducted by the primary author [27] and were based on Social Cognitive Theory. The text message content did not change during the study.

#### Control

Participants randomized to the delayed intervention were encouraged to maintain their normal eating and exercise habits for 6 months. During this 6-month period, they received 2-3 automated SMS text messages per week, which contained information on health topics specific to African American adults, including stroke prevention, lupus, cardiovascular disease, etc.

### Measures

Participants attended a baseline and 6-month visit and were required to start fasting at 10 pm the evening before each visit.

#### Outcome Measures

##### Anthropometrics

The participants’ height and weight were measured with them wearing clothes, not wearing shoes and socks, and after removing heavy pocket items. The height was measured to the nearest 0.1 cm using a wall-mounted stadiometer (Holtain Ltd., Crymych, Dyfed, United Kingdom). The weight was measured to the nearest 0.1 kg using the portable Tanita Body Composition Analyzer (SC-240).

##### Body Fat

The percent body fat was estimated using the portable Tanita Body Composition Analyzer (SC-240) [28]. Participants stood on the analyzer with their bare feet placed on the electrodes. Measurements were recorded to the nearest 0.1%.

##### Vital Signs

Blood pressure and pulse were measured using an automatic blood pressure cuff (Omron, Model BP710). Participants rested for approximately 5 minutes prior to the measurement.

##### Blood Glucose or Cholesterol

Fingerstick blood sampling (40 µL) was collected using the Alere Cholestech LDX Analyzer. Following the successful completion of quality control procedures, whole blood was collected from a fingerstick in a lithium heparin-coated capillary tube.

##### Food Intake

We used the National Cancer Institute (NCI) fat screener [29] to estimate the percentage of energy from fat over the past 12 months. The standard 7-item fruit and vegetable screener developed by the NCI and National 5 a Day Program [30,31] assessed how often fruit and vegetables were consumed in the past month.

**Session topics.**

Session 1: Obesity and chronic disease, goal setting, and self-monitoring

Session 2: Portion control, energy balance, and physical activity intensity levels

Session 3: Reading food labels, estimating calorie needs, and calorie expenditure

Session 4: Diabetes, carbohydrates, physical activity and diabetes

Session 5: Proteins, fats, physical activity and diabetes

Session 6: Cues for eating and activity behavior, and eating out

Session 7: Social support and meal planning for healthy eating

Session 8: Emotional eating and stress management

Session 9: Problem solving

Session 10: Relapse and prevention of relapse

##### Physical Activity

The International Physical Activity Questionnaire (IPAQ) [32] was used to assess self-reported physical activity. The metabolic equivalent of task (MET)-minutes/week of physical activity in each domain of leisure time activity (sedentary, light, moderate, and vigorous intensity) was calculated. The IPAQ has been shown to be a reliable and valid measure of physical activity and has been sensitive to changes in physical activity resulting from intervention programs [33].

##### Quality of Life

We used the 31-item Impact of Weight on Quality of Life Questionnaire-lite (IWQOL-lite) [34] to assess quality of life. The IWQOL-lite measures 5 domains of functioning, physical function, self-esteem, sexual life, public distress, and work. High scores indicate poor functioning.

#### Feasibility Measures

##### Text Message Tolerance

SMS text messages were deemed feasible to deliver as an intervention if <25% of participants requested a cessation of SMS text messages.

##### Satisfaction Questionnaire

Each participant’s satisfaction with the different elements of the study, including their community health coach, group sessions, and SMS text messages, was measured at the 6-month period. The questionnaire contains 15 items and is rated along a scale of Agree (1) to Disagree (5), with lower scores indicating higher levels of satisfaction. Satisfaction scores ≤2.0 (“Agree” or “Slightly Agree”) indicated the feasibility of the intervention component. The questionnaire was adapted from another satisfaction questionnaire developed by the primary author [35].

### Data Entry

All data from the measurements were entered into REDCap [36], which is a secure, Web-based application designed to support data capture for research studies, providing (1) an intuitive interface for validated data entry, (2) audit trails for tracking data manipulation and export procedures, (3) automated export procedures for seamless data downloads to common statistical packages, and (4) procedures for importing data from external sources. It improves data entry through real-time validation rules (with the automated data type and range checks) and provides easy data manipulation (with audit trails for reporting, monitoring, and querying patient records). Pennington Biomedical has a license agreement with Vanderbilt University, and the database and software are installed on our local servers and hosted by Pennington Biomedical, and not by Vanderbilt. The Vanderbilt University Office of Research was not used as a central location for data processing and management. 

### Treatment Fidelity

In this study, 2 sessions from each church were audiorecorded to allow a study staff member to monitor the intervention delivery. While the first recorded session was selected from the first 5 group meetings, the second recorded session was selected from the final 5 group meetings. The audiorecordings were reviewed and compared with a checklist of topics to be covered for each session. The topics included the introduction and closing of sessions (6 items), engaging participants (2 items), session content (7 items), and the physical activity component (5 items). Items were scored Yes (1) or No (2) for the greeting, engaging, and physical activity items, and was scored Poorly (1), Adequately (2), and Extremely Well (3) for delivery of the session content.

### Statistical Analysis

The study was a repeated measures cluster-randomized trial. Participants were nested within churches, and churches were nested within study conditions; thus, outcome data were analyzed accordingly. We used linear mixed models, with churches and participants having random effects and interventions having fixed effects, to compare the mean differences in outcome variables at baseline between the two intervention groups. The primary outcome variable was weight loss after 6 months. A second linear mixed effects model with churches having random effects and baseline outcome used as a covariate was used to analyze the outcome change between baseline and 6 months. The estimation of model parameters used restricted maximum likelihood procedures using all available data. Each secondary outcome (eg, waist circumference and questionnaires) was analyzed similarly. Multiple imputations were used to replace missing data. For each analytical mixed model, both completer’s analysis and intent-to-treat analysis were conducted to assess the sensitivity of statistical findings to our choice of statistical approach. In each analytic model, we performed a two-step procedure to test the following hypotheses: (1) churches do not have a significant effect on the outcome variable; and (2) no difference is observed in the effects of the intervention and delayed intervention. If (1) is significant, the degrees of freedom for the *F* test used in (2) depends on the number of churches, in addition to the number of participants in the churches in the intervention and control groups. If (1) is not significant, then the degrees of freedom for the *F* test used in (2) depends solely on the total number of participants. In the first case, the churches play an important role in determining the power of the test for hypothesis (1); whereas, in the latter case, the power is determined by the total number of participants. The churches vary in active membership, and although attempts were made to balance the number of participants between intervention and control groups, inequities persist between the groups because of large differences in church sizes and a small number of churches. The differential variability in the weight loss showed no evidence of a component of variability being attributable to churches, suggesting that randomization of churches effectively randomized participants to study conditions. Hence, it is feasible to assume that, except for receiving different conditions, participants in the two groups, although unbalanced, are representative samples from a common underlining population. Therefore, the validity of inferences pertaining to weight loss differences in the intervention and control groups are justified. All results are reported based on the completer’s analysis, and the results are consistent, in terms of significance, between completer’s analysis and intent-to-treat analysis, unless otherwise noted. Furthermore, Spearman correlations were calculated to assess the association between the SMS text message use and weight loss parameters. All data were analyzed utilizing SAS Version 9.4 (SAS Institute Inc., Cary, NC, USA).

### Power Analysis

The power analysis was based on recruiting 9 churches in total, with 5 churches randomized to the intervention group and 4 to the control group. The primary outcome variable was weight loss after 6 months, and the anticipated minimum difference in the average weight loss was 2 kg (~2% of the expected baseline weight). The anticipated SD for the weight loss was not expected to exceed 3.5 kg. With an alpha level set at.05, the study was designed to have at least 80% power to detect at least a 2% difference between interventions with a sample size of 12 participants in each church (total N=108).

There were 72 participants (25 from control group, 47 from intervention group) who completed the study. Fortunately, the power of the study was not compromised because the churches proved to have no differential effect on the weight loss between the study groups (see Statistical Analysis section above for the rationale). The poststudy mean weight loss was 1.6 (SD 3.1) kg, and the percent weight loss was 1.5% (SD 2.8%). These poststudy findings are more favorable than estimates used in determining power and sample size. Consequently, the poststudy power was 79%. Furthermore, secondary outcomes were viewed as possibly providing exploratory findings and therefore were not considered in power or sample size calculations.

## Results

### Demographics

Churches were recruited from February to May 2016. Initially, 9 churches were randomized. As 1 church in the delayed intervention condition was unresponsive to attempts to designate community health coaches or set a baseline date, it was subsequently dropped from the study. Therefore, 8 churches participated in this study, and the randomization resulted in 5 intervention churches and 3 delayed intervention churches. Of these, 2 churches were Catholic, 4 were Baptist, and 2 were Christian nondenominational. The churches varied in size from 75 to ~8000 members.

**Figure 1:**
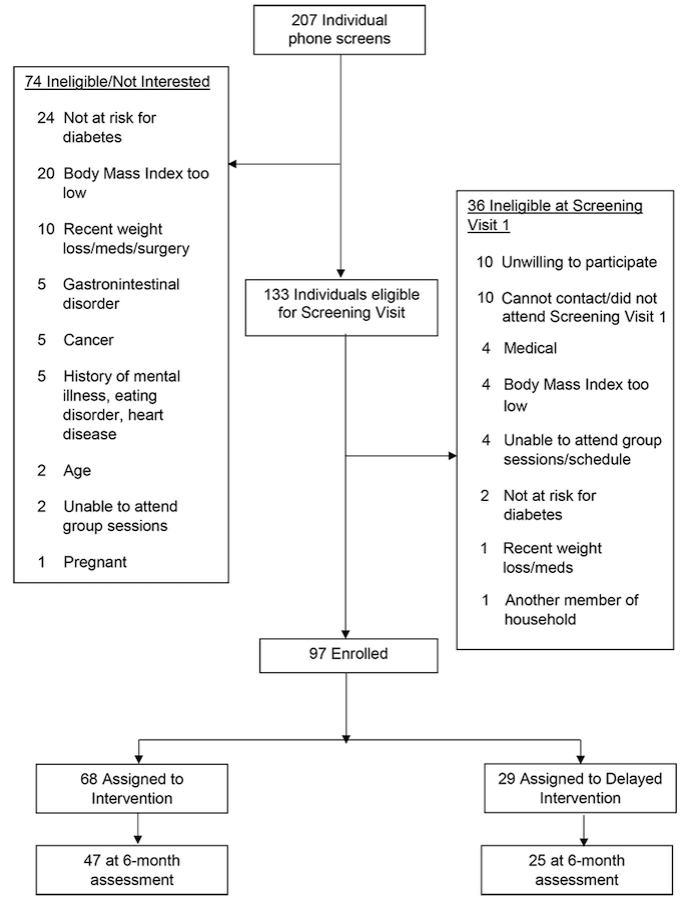
Study flowchart.

Participants were recruited from May to September 2016. We successfully recruited 97 African American adults, with a mean age of 56.0 (SE 10.3) years and a mean BMI of 38.6 (SE 6.4) kg/m^2^ (89/97, 91.8% females; 52.6% having a college or postgraduate degree), who attended churches that were randomized to the intervention (n=68) or delayed intervention (n=29) condition. In addition, 47.4% (46/97) of the sample indicated prediabetes according to the Cholestech fingerstick analysis (glucose, 100-125 mg/dL). No significant differences were noted between the study groups on baseline variables (*P*>.09; Tables 1 and 2). Notably, 74.2% (72/97) of these individuals (47/68, 69.1% intervention; 25/29, 86.2% delayed intervention) completed the 6-month assessment (Figure 1).

### Short Message Service Text Messages

Participants in both groups reported receiving SMS text messages. Although participants were not required to respond to the automated SMS text messages, 41 participants sent at least 1 text message (30 from intervention, 11 from delayed intervention). While participants in the intervention churches sent 84 SMS text messages, those in the control churches sent 25 SMS text messages, totaling 109 SMS text messages sent during the study. In addition, 24 individuals sent 80 messages that were related to the study (eg, attendance, asking for assistance, appreciation for the text, etc). However, 11 participants sent 22 messages that were determined to be unrelated to the study or were ambiguous (eg, voting, unintelligible symbols, and sleep). Furthermore, 6 individuals (4 from control, 2 from intervention) sent 7 messages requesting that the SMS text messages be stopped; this level of request (6/97, 6.1%) met our <25% feasibility criteria. Among participants in the intervention group, the correlation between the number of SMS text messages sent and change in percent body fat was statistically significant (*r*=.3 with *P*=.04), whereas the correlation between the number of SMS text messages sent and change in weight loss was not statistically significant.

### Attendance

Participants in the intervention group attended a median of 6 sessions. The average attendance rate was 55%, with attendance decreasing over the course of the intervention (*P*<.001). While session 1 had the highest attendance (70.6%), session 10 had the lowest (27.9%).

### Body Composition

A significant between-group difference was noted in weight loss (*P*=.03, effect size=0.55). Participants in the intervention lost –1.4 (SE 0.4) kg, whereas those in the control group gained 0.2 (SE 0.6) kg. No difference was observed in the percent body fat as measured by the Tanita scale (*P*=.30, effect size=0.5). While participants in the intervention lost –0.3% (SE 0.9%), those in the control group gained 1.2% (SE 1.2%; Table 3).

### Cardiometabolic Outcomes

We observed no significant between-group differences in the systolic or diastolic blood pressure, glucose, or cholesterol levels (*P*>.356).

### Quality of Life

We observed significant between-group differences on the IWQOL-lite. The total score changed significantly (*P*=.006) between the intervention and delayed intervention groups. Significant differences were observed in the physical function (*P*=.04), self-esteem (*P*=.03), and public distress (*P*=.03) subscales. A marginally significant (*P*=.08) difference was noted in the sexual life subscale. In every situation, changes indicated that quality of life improved in the intervention group and diminished in the delayed intervention group.

### Physical Activity

No significant between-group differences were observed in the change in MET-minutes/week (*P*=.99) or total time sitting (*P*=.76).

### Fruit and Vegetable Intake

We observed no significant between-group differences in the change in fruit and vegetable (*P*=.23) or percent fat intake (*P*=.41).

**Table 1:** Participants’ characteristics at baseline.

Characteristics		Control (n=29)	Intervention (n=68)	Total (N=97)
Age in years, mean (SD)		58.6 (8.7)	54.9 (10.7)	56.0 (10.3)
**Gender, n (%)**				
	Female	25 (86)	64 (94)	89 (92)
	Male	4 (14)	4 (6)	8 (8)
**Race, n (%)**				
	African American	29 (100)	68 (100)	97 (100)
**Marital status, n (%)**				
	Single	5 (17)	24 (35)	29 (30)
	Married	16 (55)	30 (44)	46 (47)
	Divorced	6 (21)	11 (16)	17 (18)
	Widowed	2 (7)	1 (2)	3 (3)
**Education, n (%)**				
	Some High School	0 (0)	2 (3)	2 (2)
	High School Diploma/General Equivalency Diploma	5 (17)	16 (24)	21 (22)
	1-3 years college	5 (17)	17 (25)	22 (23)
	College degree	7 (24)	15 (22)	22 (23)
	Post graduate degree	12 (41)	17 (25)	29 (30)
**Income, n (%)**				
	<US $50,000	11 (38)	35 (51)	46 (47)
	US $50,000- US $100,000	7 (24)	21 (31)	28 (29)
	>US $100,000	7 (24)	7 (11)	14 (14)
	Did not answer	4 (14)	5 (7)	9 (9)
Weight (kg), mean (SD)		101.6 (16.8)	103.8 (18.0)	103.2 (17.5)
Body mass index (kg/m^2^), mean (SD)		37.7 (5.6)	38.9 (6.7)	38.6 (6.4)
Body percent fat (%), mean (SD)		47.1 (6.1)	49.0 (4.7)	48.5 (5.2)
Systolic blood pressure (mmHg), mean (SD)		127.7 (14.1)	126.4 (14.3)	126.8 (14.2)
Diastolic blood pressure (mmHg), mean (SD)		75.9 (9.2)	79.9 (9.1)	78.7 (9.3)
Glucose (mg/dL), mean (SD)		112.7 (23.6)	105.2 (22.4)	107.5 (22.9)
Suspected prediabetes, n (%)		13 (50)	32 (51)	45 (51)
Total cholesterol (mg/dL), mean (SD)		177.1 (41.5)	177.7 (34.5)	177.5 (36.5)

### Medication

In this study cohort, 24 participants were taking medications to control their diabetes. One participant started taking medications after beginning the program and another decreased their dose.

### Adverse Events

No adverse events occurred during this study.

### Satisfaction Questionnaire

In this study, >80% of participants either “agreed” or “slightly agreed” with the satisfaction questions. The average satisfaction item score for the group sessions, SMS text messages, and the community health coach was 1.4 (0.53), 1.4 (0.67), and 1.4 (0.72), respectively, indicating high levels of satisfaction. These average scores of 1.4 met our feasibility criteria of <2.0.

### Treatment Fidelity

A random session from each half of the intervention was audiotaped. The average score for session content was 2.0, which is equivalent to an “adequate” rating. We conducted 40% of the actual physical activity sessions and implemented 28% of the physical activity components during the physical activity sessions. Furthermore, the community health coaches introduced and closed sessions 80% of the time and engaged participants 100% of the time.

**Table 2:** Questionnaire data at baseline.

Baseline variables		Control, mean (SE)		Intervention, mean (SE)	
**Quality of Life^a^**					
	Physical Function Scale		43.0 (1.6)		42.5 (1.0)
	Self-Esteem Scale		25.3 (1.3)		25.7 (0.9)
	Sexual Life Scale^b^		17.2 (0.8)		16.3 (0.6)
	Public Distress Scale^b^		23.0 (0.8)		22.6 (0.4)
	Work Scale^b^		17.8 (0.7)		17.7 (0.4)
	Total score		125.1 (4.3)		124.0 (2.8)
**Physical activity^c^**					
	Sitting total weekday		326.7 (51.1)		380.0 (31.9)
	Metabolic equivalent of task-min/week^b^		899.5 (249.5)		1325.3 (294.4)
**Food intake**					
	Fruits and veggies^b^		4.0 (0.5)		4.8 (0.4)

^a^Control (n=18-28), intervention (n=50-64); sample sizes differed between the different measures.

^b^Wilcoxon test, mean (SE).

^c^Control (n=27-29), intervention (n=64-68); sample sizes differed between the different measures.

**Table 3:** Six-month change scores in physiological variables.

Physiological variables		Completers analyses			Intent-to-treat analyses		
		Control (n=25), mean (SE)	Intervention (n=47), mean (SE)	*P* value	Control (n=29), mean (SE)	Intervention (n=68), mean (SE)	*P* value
**Anthropometric or physiological**							
	Percent weight loss	0.2 (0.6)	–1.4 (0.4)	.03	0.3 (0.7)	–1.6 (0.5)	.04
	Percent body fat	1.2 (1.2)	–0.3 (0.9)	.30	1.2 (1.1)	–0.4 (0.8)	.26
	Systolic	–0.3 (3.1)	0.3 (2.3)	.89	–0.4 (3.9)	0.2 (2.9)	.90
	Diastolic	0.4 (1.8)	2.4 (1.3)	.36	0.3 (2.4)	2.3 (1.8)	.51
	Glucose	–2.2 (3.9)	–6.5 (2.9)	.38	–1.8 (4.7)	–4.6 (3.4)	.64
	Cholesterol	–5.9 (6.2)	–2.4 (4.6)	.66	–6.7 (7.2)	–2.8 (5.3)	.67
**Quality of Life**							
	Physical Function	1.8 (1.2)	–1.4 (0.9)	.04	1.5 (1.3)	–1.8 (0.9)	.03
	Self-Esteem	–0.6 (1.0)	–3.4 (0.7)	.03	–0.4 (1.2)	–3.5 (0.8)	.03
	Sexual Life	0.4 (0.7)	–1.1 (0.5)	.08	0.8 (0.9)	–1.0 (0.6)	.11
	Public Distress	0.2 (0.4)	–0.8 (0.3)	.03	0.2 (0.4)	–0.8 (0.3)	.04
	Work	–0.2 (0.4)	–0.3 (0.3)	.80	–0.03 (0.5)	–0.6 (0.4)	.38
	Total	1.8 (2.5)	–6.9 (1.8)	.01	2.9 (3.1)	–7.4 (2.4)	.01
**Physical activity**							
	Sitting total weekday	5.7 (50.2)	–13.1 (33.7)	.76	–1.7 (41.1)	–12.5 (28.9)	.82
	Metabolic equivalent of task-min/week	586.4 (503.3)	587.2 (362.8)	.99	373.5 (492.9)	295.8 (335.5)	.90
**Food intake**							
	Fruits and veggies	0.8 (0.7)	–0.3 (0.5)	.23	0.8 (0.7)	–0.2 (0.5)	.27
	National Cancer Institute percent fat	1.4 (0.6)	2.1 (0.4)	.41	1.67 (0.6)	2.0 (0.5)	.63

## Discussion

### Principal Findings

The LEAN study showed that church-affiliated African American participants were receptive to receiving SMS text messages related to behavioral strategies as part of a weight loss intervention. Participants engaged with SMS text messages, indicated satisfaction with the SMS text messages, and few requested the messages to be stopped, demonstrating the feasibility of the intervention. This church-based study resulted in statistically significant weight loss and improved quality of life for participants in the intervention group compared with the control group. Importantly, engagement with SMS text messages was associated with body fat loss. However, other variables assessed, including self-reported physical activity and dietary intake, and clinical outcomes, were unchanged.

The unique component of the LEAN study was the use of SMS text messages in a randomized controlled trial targeting African American adults. Three recent studies of African American adults have utilized SMS text messages to assist in the initiation of behavioral change in a church-based setting [25,26,37]; the SMS text messaging system in these studies included personalization, support, and delivering recipes and meal plans. They also showed within-group changes in weight loss or physical activity outcomes across 12 weeks. The LEAN study builds upon these investigations by utilizing a randomized controlled trial design and intervening over a 6-month period. The use of SMS text messages in the LEAN study did not appear to result in greater weight loss compared with most other church-based studies targeting African American adults [38]. However, our satisfaction data showed that SMS text messages were well-received, similar to recent studies [25,26,37]. Of note, the majority of LEAN participants responded to SMS text messages even though they were not required to do so, and higher text message response was associated with higher percent body fat loss. It is difficult to explain the correlation between text message response and body fat loss in the absence of a significant correlation with BMI. Although BMI and percent body fat are highly correlated, these do not measure the same component of adiposity. Participants responding to SMS text messages might have made behavioral changes that affected bodily tissues (eg, adipose tissue size and water) that are estimated through body impedance but not BMI. Alternatively, this could also be a spurious finding, and therefore, inferential interpretation should be made with caution until this finding can be replicated. Nonetheless, the LEAN study demonstrates that it is feasible and potentially efficacious to incorporate a form of mHealth (SMS text messages) into a translational behavioral change study targeting African American adults. It is suggested that future health disparities research targeting African American adults should explore the use of other mHealth technologies, including apps, social networking, and wearables [39,40]. These mHealth technologies could provide objective assessments of physical activity, diet, and weight; immediate feedback on participant behavior; automated or personalized messages (eg, from the church-leadership or lay health provider); and more frequent and effective communication between lay health providers and participants, which may enhance efficacy. Future work should also utilize innovative methodologies such as optimization, microrandomized trials, and adaptive designs [41-43].

### Comparison With Prior Work

The LEAN study is one of the numerous church-based translational weight loss programs targeting African American adults. Lancaster et al [38] recently conducted a systematic review of these programs showing that the majority of studies reported statistically significant weight loss, similar to the LEAN results. The LEAN study incorporated elements common among the studies reviewed, including enrolling overweight or obese African American adults, embedding religious principles within the intervention, and utilizing lay health advisors (known as “community health coaches” in the LEAN study) to deliver the intervention. However, few studies achieved clinically significant weight loss of >3% [26,38,44], including the LEAN study. Our treatment fidelity data indicated that of the subset of sessions observed, the physical activity component was infrequently implemented, and although the actual weight loss content was delivered adequately, there was room for improvement. Enhancing the community health coach training or increasing contact with the community health coaches during the implementation of the project through booster sessions might improve the program implementation adherence. Another contributor to the small weight loss might be the lack of attendance. Greater weight loss in the LEAN study could have been obtained through a combination of enhanced implementation and greater attendance.

Few church-based weight loss interventions have assessed quality of life [26,45]. Some similar church-based health promotion interventions involving individuals with overweight or obesity have measured overall quality of life but did not measure the impact of participants’ weight on their quality of life [26,44]; this is an important aspect for individuals with overweight or obesity. Kennedy et al [45] utilized the IWQOL scale but did not find changes in their church-based weight loss intervention. The LEAN findings suggest that the intervention decreased the negative impact that weight had on the participants’ quality of life. As quality of life is an important outcome from a patient-centered perspective, it is necessary to continue to investigate the effect of church-based weight loss studies on this outcome.

### Strengths and Limitations

There are several strengths of the LEAN study. The LEAN study is one of the few church-based programs to incorporate text messaging, utilize a cluster-randomized trial design, and include a delayed intervention control group. It was delivered in 8 different churches, included assessment of treatment fidelity and was adequately powered to detect changes in the primary outcome variable. In addition, the LEAN study included a number of measures beyond weight loss, including an estimated percent body fat, cardiometabolic outcomes, impact of weight on quality of life, and treatment satisfaction. However, this study also has several limitations. First, the sample sizes were unbalanced between the intervention and control groups, which could be attributed to the fact that we were able to recruit ~14 participants per church in the intervention condition and only ~9 per church in the control condition. Nevertheless, sufficient power remained to detect significant differences. In addition, the LEAN study provides important findings related to the feasibility of mHealth interventions in this practical setting, and efficacy can be confirmed in a larger clinical trial. Second, the LEAN study was only 6 months in duration. However, the duration was twice as long as similar studies [25,26,37] and was sufficient to determine the study feasibility. Third, in terms of mHealth technology, automated, one-way SMS text messages are rather rudimentary. The fact that SMS text messages were not personalized, or that SMS text messages did not provide feedback on participants’ behavior, might have contributed to the small weight loss findings. However, one of the purposes of this study was to determine whether automated SMS text messages were feasible to deliver to this population, and we were able to achieve this aim. In addition, even these simple SMS text messages were associated with percent body fat loss. Fourth, while the LEAN study comprised participants with some demographic diversity (education, income, marital status, etc), it will be important to show that the study can be effective in different regions of the United States and with a greater proportion of male participants. Finally, we utilized self-report measures of diet and physical activity.

### Conclusions

Overall, the LEAN study demonstrated that mHealth technology (ie, SMS text messages) is feasible to deliver and well-received by African American adults in a church-based setting. The study also showed that a church-based intervention resulted in significant weight loss and improvements in quality of life. A key to future investigations will be to determine whether more advanced mHealth technologies (eg, mobile apps, activity monitors, and automated scales) can be implemented successfully, without increased burden on the lay health provider or participants. Furthermore, it will be important to determine whether these technologies can increase weight loss to clinically significant levels and to show that mHealth technology can be utilized over an extended period.

## Abbreviations

BMI: body mass index
IPAQ: International Physical Activity Questionnaire
IWQOL-lite: Impact of Weight on Quality of Life Questionnaire-lite
LEAN: Lifestyle Changes Through Exercise and Nutrition
MET: metabolic equivalent of task
NCI: National Cancer Institute
SMS: short message service

## Appendix

Multimedia Appendix 1 CONSORT EHEALTH checklist (V 1.6.1).
